# Association of polymorphisms rs1800012 in COL1A1 with sports-related tendon and ligament injuries: a meta-analysis

**DOI:** 10.18632/oncotarget.15271

**Published:** 2017-02-11

**Authors:** Chunguang Wang, Hao Li, Kang Chen, Bing Wu, Haifeng Liu

**Affiliations:** ^1^ Department of Sports Medical, The First Affiliated Hospital of Shenzhen University, Shenzhen Second People's Hospital, Futian District, Shenzhen, Guangdong, China

**Keywords:** COL1A1 polymorphism, rs1800012, sports injury, tendon and ligament injury, ACL injury

## Abstract

It has been reported that the single nucleotide polymorphism (SNP) rs1800012 in COL1A1 might be associated with the susceptibility to sports-related tendon and ligament injuries such as ACL injuries, Achilles tendon injuries, shoulder dislocations and tennis elbow. But the data from different studies have been conflicting. Here we attempted to systematically summarize and clarify the association between the SNP and sports-related tendon and ligament injuries risk. Six eligible studies including 933 cases and 1,381 controls were acquired from PubMed, Web Of Science and Cochrane library databases. Significant association was identified in homozygote model (TT *versus* GG: OR=0.17, 95%CI 0.08-0.35, *P*_H_=0.00) and recessive model (TT *versus* GT/GG: OR=0.21, 95%CI 0.10-0.44, *P*_H_=0.00). Our results indicated that COL1A1 rs1800012 polymorphism may be associated with the reduced risk of sports-related tendon or ligament injuries, especially in ACL injuries, and that rare TT may played as a protective role.

## INTRODUCTION

With the improved quality of life, increasingly sports demands were raised, and growing individuals are suffering from sports injuries, which is estimated of a 15.47 per 1,000 incidence in NCAA athletes [1]. Sports injuries occurred all around us while tendon and ligament injuries constitute the most. [2]

Tendons and ligaments are collagenous structures which is made up of type I collagen fibrils. [3, 4] And COL1A1 encoding the Type I collagen, is indicative of a relationship between them. Actually, there do be plenty of literatures that reporting the association of COL1A1 with tendon and ligament injuries susceptibility. Single nucleotide polymorphisms (SNPs), occurring in the COL1A1 gene region, may alter the COL1A1 expression and affect the property of COL1A1 and subsequently lead to the susceptibility to injury. Among various polymorphisms within the COL1A1 gene, the most frequently studied polymorphism has been the +1245G/T polymorphism (rs1800012, Sp1), it is a G to T polymorphism lying within the first intron of COL1A1 affecting a binding site for the transcription factor Sp1. [5, 6]

A multiple of diseases can have a genetic disorder, some are the Mendelian inherited conditions while the others are non-Mendelian inheritance spectrum such as tendon and ligament injuries, which are results from multifactorial issues and the severity of the conditions are determined by the complex gene-gene and gene-environment interactions [7]. Studies focus on identifying the association of genetic mutations with tendon and ligament injuries are of important significance and could help to predict injury risk for specific individuals or guide the clinical management of ‘high-risk’ individuals [8, 9]. Although several articles have been reported on the association between rs1800012 and the risk of tendon and ligament injuries in diverse population, the results were mixed and inconclusive. Up to now, there is no meta-analysis investigating the association between them. Therefore, we performed a meta-analysis to evaluate the association between rs1800012 and tendon and ligament injuries risk.

## RESULTS

### Characteristics of studies

**Figure 1:**
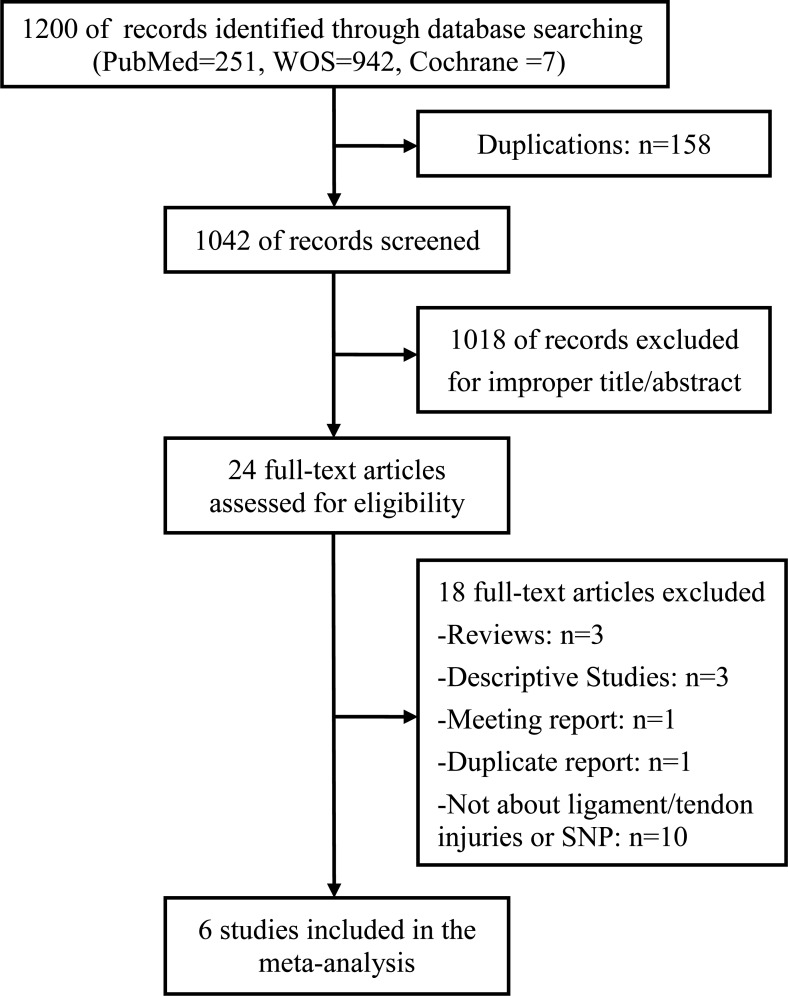
Flow diagram of studies identified, included, and excluded

A total of 1200 studies were acquired from PubMed, Web Of Science and Cochrane library databases (PubMed = 251, WOS = 942, Cochrane = 7). Six studies [10–15] containing 933 cases and 1,381 controls fulfilled the predefined inclusion criteria and were included in the final analysis. The literature selection process was shown in Figure 1. And the characteristics of included study were shown in the Table 1. All the subjects in the included studies were of Caucasian population. Among the six studies, there were three anterior cruciate ligament (ACL) injuries studies, one Achilles tendon injuries study, one Tennis elbow study and one shoulder dislocations and cruciate ligament (CL) injuries study. All the ACL injuries or CL injuries were surgically diagnosed and the shoulder dislocations were radiologically confirmed. Tennis elbow injuries were diagnosed using clinical criteria while the Achilles tendon injuries including the diagnoses of chronic Achilles tendinopathy and the partial or complete ruptures of the Achilles tendon. All the participants in the control groups were healthy participants without any history of ligament or tendon injuries. The genotyping distribution was in agreement with HWE in all studies.

**Table 1:** Characteristics of studies included in the meta-analysis of COL1A1 rs1800012 G>T and sports injuries risk

Study ID	Year	Country	Ethnicity	Diagnosis	Case			Control			P for HWE	Quality
					GG	GT	TT	GG	GT	TT		
Khoschnau et al.	2008	Sweden	Caucasian	All Cruciate ligament ruptures Shoulder dislocations	257 162 95	99* 70 30	2 1 1	230	83	12	0.20	*****
Posthumus et al.	2009a	South Africa	Caucasian	ACL injuries	80	37	0	91	33	6	0.20	*****
Posthumus et al.	2009b	South Africa	Caucasian	Achilles tendon injuries^#^	91	33	2	89	31	6	0.14	*****
Ficek et al.	2013	Poland	Caucasian	ACL injuries	65	26	0	96	41	6	0.55	******
Stepien-Slodkowska et al. Erduran et al.	2013 2014	Poland Turkey	Caucasian Caucasian	ACL injuries Tennis elbow	90 69	46 32	2 2	139 61	39 35	5 7	0.27 0.52	****** ******

* 1 patient had both diagnoses

^#^chronic Achilles tendinopathy and ruptures of the Achilles tendon

### Association between COL1A1 rs1800012 polymorphism and sports-related tendon and ligament injuries susceptibility

**Figure 2:**
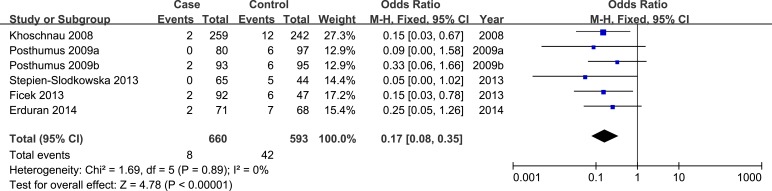
Forest plot of homozygote model of COL1A1 rs1800012 for overall comparison (TT versus GG)

**Figure 3:**
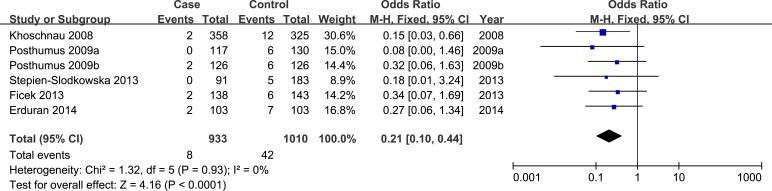
Forest plot of recessive model of COL1A1 rs1800012 for overall comparison (TT versus GT+GG)

In allele model, random-effects model was used due to presence of heterogeneity. No significant heterogeneity was identified by I-squared statistic in any of the rest genetic models and thus fixed-effects model was used. Overall, significant association was identified in homozygote model (TT *versus* GG: OR = 0.17, 95%CI 0.08-0.35, *P*_H_ = 0.00 Figure 2) and recessive model (TT *versus* GT/GG: OR = 0.21, 95%CI 0.10-0.44, *P*_H_ = 0.00 Figure 3). No significant association was found in allele model (T *versus* G: OR = 0.89, 95%CI 0.75-1.05, *P*_H_ = 0.17), heterozygote model (GT *versus* GG: OR = 1.13, 95%CI 0.93-1.38, *P*_H_ = 0.23), and dominant model (TT/GT *versus* GG: OR = 1.00, 95%CI 0.83-1.22, *P*_H_ = 0.97). (see Table 2)

**Table 2:** Summary of polled ORs in the meta-analysis

rs1800012	N	OR	P_H_	OR	P_H_	OR	P_H_	OR	P_H_	OR	P_H_
		T/G		GT/GG		TT/GG		TT+GT/GG		TT/GT+GG	
Overall	6	0.89 (0.75-1.05)	0.17	1.13(0.93-1.38)	0.23	0.17 (0.08-0.35)	0.00	1.00 (0.83-1.22)	0.97	0.21 (0.10-0.44)	0.00
Diagnosis											
ACL injuries	3	1.02 (0.78-1.33)	0.90	1.34(0.98-1.83)	0.07	0.23 (0.07-0.79)	0.02	1.19 (0.87-1.61)	0.27	0.21 (0.06-0.73)	0.01
CL injuries	1	0.93 (0.67-1.28)	0.65	1.20(0.82-1.74)	0.35	0.12 (0.02-0.92)	0.04	1.06 (0.73-1.53)	0.75	0.11 (0.01-0.87)	0.04
Achilles tendon	1	0.84 (0.52-1.35)	0.46	1.04(0.59-1.84)	0.89	0.33 (0.06-1.66)	0.18	0.93 (0.54-1.60)	0.78	0.32 (0.06-1.63)	0.17
Shoulder	1	0.74 (0.48-1.13)	0.16	0.88(0.54-1.42)	0.59	0.20 (0.03-1.57)	0.13	0.79 (0.49-1.26)	0.33	0.21 (0.03-1.62)	0.13
Tennis elbow	1	0.68 (0.42-1.10)	0.11	0.81(0.45-1.46)	0.48	0.25 (0.05-1.26)	0.09	0.72 (0.41-1.26)	0.25	0.27 (0.06-1.34)	0.11

**Figure 4:**
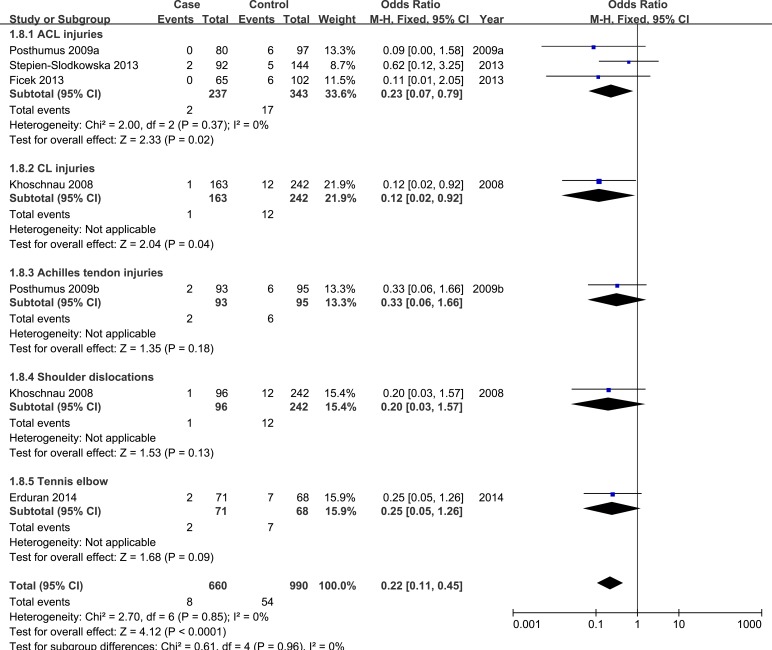
Forest plot of homozygote comparison of COL1A1 rs1800012 for subgroup analysis stratified by diagnosis (TT versus GG)

**Figure 5:**
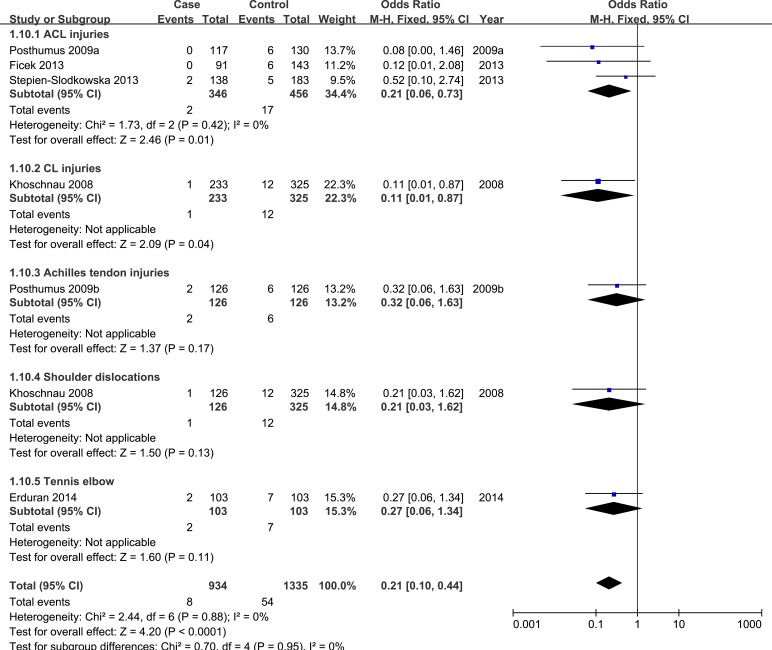
Forest plot of recessive comparison of COL1A1 rs1800012 for subgroup analysis stratified by diagnosis (TT versus GT+GG)

Next, subgroup analysis was conducted according to different diagnoses. The association between COL1A1 rs1800012 polymorphism and the risk of ACL injuries was analyzed in three independent studies. In ACL injuries, no significant statistical heterogeneity was identified in any genetic model so that fixed-effects model was used. Significant association was found in homozygote model (TT *versus* GG: OR = 0.23, 95%CI 0.07-0.79, *P*_H_ = 0.02 Figure 4), and recessive model (TT *versus* GT/GG: OR = 0.21, 95%CI 0.06-0.73, *P*_H_ = 0.01 Figure 5). No significant association was found in allele model (T *versus* G: OR = 1.02, 95%CI 0.78-1.33, *P*_H_ = 0.90), heterozygote model (GT *versus* GG: OR = 1.34, 95%CI 0.98-1.83, *P*_H_ = 0.07), and dominant model (TT/GT *versus* GG: OR = 1.19, 95%CI 0.87-1.61, *P*_H_ = 0.27). (see Table 2)

### Sensitivity analysis

Sensitivity analysis was performed to examine the influence set by the individual study on the pooled ORs for COL1A1 rs1800012 polymorphism by deleting one study each turn in every genetic model. [16] There was no change in the significance of any of the outcomes except for the allele model, in which the pooled estimate showed statistical difference after excluding the study of Stepien-Slodkowska [15] (T *versus* G: OR = 0.81, 95%CI 0.67-0.98, *P*_H_ = 0.03).

## DISCUSSION

This meta-analysis of six eligible case-control studies including 933 cases comparing 1381 healthy controls showed that rare TT genotype was likely a protective factor for sports-related tendon and ligament injuries, as the odds ratio was 0.17 in the homozygote model and 0.21 in the recessive model respectively. In subgroup analysis, similar results were found when the analysis was limited to ACL injuries. However, this finding should be interpreted with caution for limited sample heterogeneity.

Type 1 collagen, representing up to 80% of the dry weight of tendon and ligament tissue [17], is composed of two chains: α1 and α2, at a ratio of 2 : 1. The COL1A1 gene encodes for the α1 chain and a polymorphism of this gene may affect the gene transcription, leading to a change in the α1/α2 ratio, then generating a mature collagen protein with an altered structure. [5, 18] Among the various SNPs, rs1800012 is the most studied one and has been associated with connective tissue disorders such as osteogenesis imperfecta [19], osteoarthritis [20], and soft tissue injuries in a combined analysis [21]. Previous studies showing a significant genotypic association of COL1A1 rs1800012 polymorphism TT genotype with tendon and ligament injuries can be found in a Sweden population [12] and a Poland population [11], although this finding could not be replicated by another Poland population study [15]. No significant association of rs1800012 SNP was noted in two South Africa works [13, 14] and a Turkey examine [10], as well as a none-Caucasian study [22]. By the way, there is a second COL1A1 polymorphism (COL1A1 1997G/T, rs1107946) that has been studied with the incidence of ACL injuries, whereas no significant association was identified. [11, 23]

Our meta-analysis has several strengths. In the first place, this is the first meta-analysis focused on the association between COL1A1 polymorphism and the susceptibility to sports-related tendon and ligament injuries. Compared with the former review regarding ACL injury gene etiology [24], more COL1A1 studies were included and more systematic and specified meta-analysis was performed. Moreover, multiple strategies and strict criteria were used to evaluate the methodological quality of the studies, all the enrolled studies possessed high qualities and no study was calculated significant departure from Hardy-Weinberg equilibrium (HWE). The last but not the least, no significant heterogeneity was detected in any genetic models, thus our meta-analysis results enjoys high credibility.

The present meta-analysis has the following limitations that must be taken into account. Firstly, the number of included studies for COL1A1 rs1800012 polymorphism limited further analysis due to shortage of original studies. Secondly, the cases enrolled in this study are from different sports groups, some are skiers, some are soccer players, while some others were not mentioned. To our knowledge, sports-related tendon and ligament injuries have both intrinsic and extrinsic risk factors [25], characters from different sports groups may lead an influence to the final results. Thirdly, in one of the enrolled studies [12], two different injuries cases were compared to the same control group, then the control number in the subgroup analysis was a little larger than it really is, resulting in the subgroup analysis results being treated with cautionary attention. What's more, all the including studies were Caucasian ethnicity, more studies basing on other ethnicities remained to be performed.

In conclusion, our results indicated that COL1A1 rs1800012 polymorphism may be associated with the reduced risk of sports-related tendon or ligament injuries, especially in ACL injuries, and that rare TT may played as a protective role. Nevertheless, despite our rigorous methodology, the inherent limitations of included studies and insufficient data prevent us from definitive confirming the association of sports-related tendon or ligament injuries and rs1800012 in COL1A1. Future studies with large-volume and more ethnic groups are awaited to validate and update the findings of this analysis.

## METHODS AND MATERIALS

### Identification of eligible studies

A literature search was performed in Pubmed, Web Of Science and Cochrane Library databases on June 2, 2016, without restriction to regions, publication types, or languages. We used the following search terms in [Title/Abstract]: [Collagen Type I Alpha I OR Collagen Type I Alpha1 OR Collagen Type1 Alpha 1 OR Collagen Type I α1;1 OR Collagen Type 1 α1;1 OR Type 1 Collagen α1;1 OR Type I Collagen α1;1 OR Col1a1 OR COL1A1 OR COLIA1] AND [muta* OR varia* OR polymorphi* OR SNP] AND [Wounds and injuries OR injur* OR trauma* OR sprain* OR strain* OR fracture* OR bone broke OR dislocation* OR tendinopathy OR luxation* OR contusion* OR tendon* OR muscle tear OR rupture*]. The reference lists of all retrieved studies, review articles, and conference abstracts were also manually searched for further relevant studies.

### Inclusion and exclusion criteria

Virtually all common sports injuries occurred in the shoulder, elbow, knee or ankle joint. Sports injuries which will be covered in this meta-analysis include the rotator cuff tendon injuries in the shoulder (rotator cuff injuries), tendon injuries in the elbow (tennis elbow), the cruciate ligament injures in the knee (cruciate ligament injuries), and Achilles tendon injuries in the ankle (Achilles tendon injuries). Studies in this meta-analysis must also meet the following inclusion criteria: (1) evaluation of the association between COL1A1 polymorphisms and sports injuries; (2) case-control study; (3) studies focusing on human being; (4) detailed genotype data could be acquired to calculate the odds ratios (ORs) and 95% confidence intervals (CIs); Exclusion criteria: (1) duplication of previous publications; (2) comment, review and editorial; (3) family-based studies of pedigrees; (4) study with no detailed genotype data. When there were multiple publications from the same population, the most recent or complete study was included. Eligible studies was achieved by two contributing authors independently in accordance with the inclusion and exclusion criteria by screening the titles, abstracts and full-text. Any disagreement was resolved by discussions.

### Data extraction

Data from the included studies were extracted and summarized independently by two of the authors (Li and Chen). Any disagreement was resolved by the adjudicating senior authors (Liu and Wang). The contents listed below were collected: name of first author, year of publication, the characteristics of cases and controls, country of origin, the detective sample, ethnicity, genotyping methods, the criteria of injury, Hardy-Weinberg equilibrium, number of cases and controls, genotype frequency in cases and controls.

### Methodological quality assessment

Studies were rated for the level of evidence provided according to the Newcastle-Ottawa scale [24, 26], which consists of three factors: study selection (4 items; 1 star can be awarded for each item), comparability of cases (2stars can be awarded), and exposure (2 items; 1 star can be awarded for each item). A score of 0-8 (allocated as stars) was allocated to each study and a high score means excellent quality of the study. Two investigators scored the studies independently and solved disagreement through discussion.

### Statistics analysis

We conducted our meta-analysis following preferred reporting items for systematic reviews and meta-analyses (PRISMA) statement [27]. We evaluated Hardy-Weinberg equilibrium (HWE) for each study, and *P* < 0.05 was considered a significant departure from HWE. OR and 95% CIs were calculated to evaluate the strength of the association between COL1A1 SNP and susceptibility to sports injuries. Pooled ORs were performed for allelic comparison (T *versus* G), heterozygote model (GT *versus* GG), homozygote model (TT *versus* GG), dominant model (TT + GT *versus* GG ), recessive model (TT *versus* GT + GG), respectively. A statistical Z-test was used and P value less than 0.05 was taken to indicate statistical significance. Statistical heterogeneity was evaluated using Cochran Q tests and the I^2^ statistic (greater than 50% as evidence of significant inconsistency) [28]. The random-effects model was used if there was heterogeneity between studies; otherwise, the fixed-effects model was used. Subgroup analyses were carried out on stratification of different injury types. Sensitivity analyses were performed to evaluate the effect of each study on the combined ORs. All statistical analyses were performed using Review Manager 5.3 (Cochrane Collaboration, Oxford, UK).
